# Cast Aluminum

**Published:** 1894-09

**Authors:** 

**Affiliations:** Chicago


### COMMUNICATIONS.

### Cast Aluminum.

REMARKS BY DR. HARPER, OF CHICAGO.

Before the 38th Annual Session of the Michigan Dental Association.

GENTLEMEN :—In demonstrating to you the Zeller Cast Aluminum Apparatus, I do so with the fullest confidence in it, and also with the assurance of a perfect plate when I open my flask after casting.

After much experience with it and other apparatus for this work, I unhesitatingly state that it is the only successful method at present before the profession. I do not mean to infer that plates cannot be cast by any other process, but it has been my experience to find that with other methods there is an average of at least one failure in ten—this, too, in the hands of an expert. With the Zeller, a man of ordinary skill can depend upon a perfect plate always.

In a successful method for casting aluminum, much is dependent upon the metal, and the Zeller alloy meets every requirement. The cast will be found clean and smooth enough to be placed in the mouth without polishing, if preferred so, as will be found in the cast made at this demonstration.

The shrinkage in the Zeller alloy is extremely small, permitting the operator to cast to plain teeth without fear of a check or imperfection. (This will be shown in the plate to be cast at this meeting.)

The tendency of pure aluminum to crystallize, with results in a very rough plate, is entirely overcome in this metal.

The metal is totally free from chemical action in the mouth. This has been proven by four years wear, and also by tests made in various solutions out of the mouth. Plates have been immersed for a period of seventy-two hours in the following solutions:

One teaspoonful of salt to one pint of water.
One tablespoonful of salt to one pint of water.
Equal parts of acetic acid and water.
Full strength of acetic acid and water.

There was absolutely no sign of chemical action and the solutions failed to give a test for aluminum by addition of the proper re-agents.

Mitchell's Dental Chemistry states: "Aluminum is soluble in hydrochloric acid and aqueous solutions of alkaline hydrates and resists cold acids, mineral and vegetable, except hydrochloric;" and my own experiments confirm this.

Plastic amalgam cannot be worked in contact with cast aluminum, particularly in an alkaline saliva, as the mercury combines with it superficially, and this in turn, when exposed to the air, takes up oxygen and forms alumina which appears on the surface as a white excrescence. Fillings of amalgam which have crystallized or hardened have no effect on cast aluminum, and a cast plate or bridge may be placed in contact with them with perfect safety.

To give a better understanding of this method I will describe it briefly from the taking of an impression to the finished plate, and this as I proceed in the casting of the case before you.

The impression (which I always prefer in plaster) should be as smooth and perfect as possible. I scrape it slightly from just behind the center of the alveolar ridge to within a quarter of an inch of the posterior margin of the plate (the same as for other plate work). I now paint it with a thin solution of shellac varnish and set aside for at least thirty minutes (this to allow it to set perfectly). I now coat the surface of the impression, except on the buccal surface of the alveolar ridge posterior to the cuspids, with a thin solution of sandarach varnish.

I have found by actual measurement that the shrinkage across the heel of a plate amounts to about $\frac{1}{120}$ of an inch, and this is enough to make the plate fit snugly, and, if increased, would prevent the plate from coming in contact with the hard palate and thus interfere with the suction. I have found that the two coats of sandarach, if the impression is covered on the buccal sides mentioned, amounts in thickness to more than the shrinkage of the metal, and for this reason is objectionable, as it makes the plate too narrow across the heel, bringing all the pressure on the buccal surface of the alveolar ridge and preventing contact in the region of the hard palate.

After coating with sandarach as described, I allow it to stand again for at least thirty minutes, after which time I dip a camel-hair brush in dry pulverized soapstone and rub over the surface of the impression; then I run my model (using for this ground asbestos and plaster of paris) and set aside for about two hours.

In cases where there is no undercut, it will be found that by careful tapping on the bottom of the impression they will separate without destroying either the impression or model, and the surface of the latter will be found smooth and polished, much the same as polished marble. If there are any undercuts, I break away the buccal or labial portion of the impression and then separate.

The above in detail is the best method I have found for making a perfectly smooth model, which is so essential in cast aluminum work.

I next wax up the case as desired, using wax about the thickness of the ordinary pink rubber, or thinner, which can be obtained at the dental depots.

The case is now invested in a Zeller flask. This differs from all others used for this purpose, in that the crucible is cast to it; also, that it has on each side of the crucible two air-escape holes which communicate with the interior of the mold by means of circular and lateral canals. In these holes is placed a piece of fine wire gauze which permits the escape of the air but checks the metal so that it can be cast under pressure.

The case, being flasked, is now placed in the apparatus and dried out slowly, which usually takes about one hour. This brings me to a very important point in cast aluminum work.

You will notice that the apparatus is constructed with two burners, each of which is furnished with an air tube for connection with the blower. The interior of the lower burner is the same shape as the exterior of the flask, except that it is a little larger, just enough to allow the flame to reach it at the point where combustion is most complete. By this means the flask is surrounded by a uniform heat, which may be regulated as desired, and will permit the operator to make it, with contents, white hot in five minutes by means of the blast. This is one of the most essential points in cast aluminum work and one that is overlooked in other apparatus.

As soon as the flask is perfectly dry I connect the blower with the lower burner and continue with the blast until the flask and contents are a bright red heat. I maintain this for a period of at least five minutes; this latter is absolutely necessary, as the contents of flask (asbestos) is so poor a conductor and requires time to absorb the heat so necessary to a perfect cast.

Being satisfied that the mold has acquired this heat I now turn off the gas from the lower burner for a period of five minutes (exactly). I now drop an ingot of metal in the crucible and turn on the gas in the upper burner, which you will notice is round and adapted to the circumference of the crucible, the same as the flask burner and for the same purpose. When the above-mentioned time—five minutes—has elapsed, I ignite lower burner with low flame, and without blast (this is simply to hold flask and contents at this temperature, and experience has demonstrated it to work best at this heat) and proceed to fuse the aluminum by means of the blast being directed around the crucible. I never allow the crucible to become white hot, as there is great danger of burning the metal which would result in a spongy plate The metal is watched closely, and as soon as it is noticed to be completely fused I insert the two brass tubes, as shown, into the air-escape holes at side of the crucible, giving them about ten seconds to absorb heat and expand to fit air-tight in the holes.

I now make my cast by creating a vacuum within the mold. This I find is done best by taking *one* long and strong inhalation with the lungs. You will notice as the air is taken out of the mold, atmospheric pressure forces the metal in, and a perfect cast is the result. I now place the cap on top of crucible and submit to pressure from the lungs, merely to make a more dense and smoother cast. It is now set aside to cool, after which it is trimmed and filed to fit the mouth.

Many prefer to place the plate as cast in the mouth. In my practice I burnish, and this is best done with a gold-finishing bur, one about the size of a pea, in the dental engine. If the plate should be cast rough in spots, I grind smooth with a small corundum wheel, and then burnish as before mentioned, always using oil for this purpose. I now finish with brush wheels and wet pulverized pumice stone and finally with a dry wheel or dry felt cone—the brush making a bluish finish, the cone a white.

I have little to say of the merits of cast aluminum work, as the sample cases I present for your inspection are sufficient testimony.

The exceedingly slight shrinkage of the Zeller alloy makes a plate which is unsurpassed for adaptation and perfect suction, as will be best understood by the following which I quote from Essig's work on metallurgy:

"Plaster expands on setting. From the impression to the model, two expansions are gone through before the fac-simile of the mouth in plaster is obtained; hence, a plate made to fit such a model perfectly must necessarily be somewhat larger than the mouth—a condition unfavorable to atmospheric adhesion; better results might be expected where the plate is somewhat smaller than the mouth, because such a condition would, in entire upper dentures, throw any under-pressure upon the alveolar ridge, while that portion of the plate covering the palatine arch would barely be in contact with the tissues; the pressure along the ridge would quickly promote absorption of the remains of the alveoli, and a uniform adaptation of the plate to the mouth would soon follow.

"On the contrary, if the plate be made to fit the plaster cast, and is a trifle larger than the mouth, the pressure will be thrown upon the palatine arch at the back edge of the plate at a region not likely to change by absorption, as is the case with the alveolar ridge, and hence the margin of the plate will imbed itself in the tissues and cause much discomfort and impair the usefulness of the denture."

In attaching teeth by rubber before flasking to vulcanize, I paint the plate with sandarach varnish, except where the rubber is attached; this prevents any discoloration which would occur if unprotected.

